# Vitamin D status in critically ill patients: the evidence is now bioavailable!

**DOI:** 10.1186/cc13975

**Published:** 2014-07-07

**Authors:** Gennaro De Pascale, Sadeq A Quraishi

**Affiliations:** 1 Department of Anesthesia, Critical Care and Pain Medicine, Massachusetts General Hospital, Harvard Medical School, 55 Fruit Street, Boston, MA 02114, USA; 2 Department of Intensive Care and Anesthesiology, Agostino Gemelli Hospital, Catholic University of the Sacred Heart, L.go A. Gemelli 8, Rome, 00168, Italy

## 

We read with great interest the recent article by Amrein and colleagues demonstrating an association between 25-hydroxyvitamin D (25(OH)D) levels and adjusted hospital mortality (hazard ratio 2.05; 95% confidence interval 1.31 to 3.22) [1]. Interestingly, in this large cohort of ICU patients, no individual with 25(OH)D levels ≥30 ng/ml died from sepsis. However, the investigators did not appreciate an association of 25(OH)D levels with hospital length of stay, ICU mortality, blood culture positivity, or inflammatory markers. We hypothesize that these negative findings may be explained by the fact that only total serum 25(OH)D levels were available for the analysis.

Circulating 25(OH)D levels are considered the best indicator of vitamin D status in the general population [2]. However, 25(OH)D is predominantly bound to vitamin D binding protein in a very stable complex; indeed, only free and albumin-bound 25(OH)D may be considered available for biological functions [3]. Formulas used to calculate the bioavailable fraction, however, were not derived from critically ill patients and may be inadequate in the ICU setting. In addition, expression of human cathelicidin, a potent vitamin D-dependent antimicrobial peptide, is suppressed in the setting of low 25(OH)D levels [4]. Moreover, how bioavailable 25(OH)D and cathelicidin expression change over the course of critical illness remains unclear [5].

Hence, we strongly urge future studies related to vitamin D status in critical illness to consider incorporating direct measurements of bioavailable 25(OH)D and cathelicidin expression to further our understanding of the potential underlying biological mechanisms by which vitamin D optimization may improve ICU outcomes.

## Authors’ response

Karin Amrein, Paul Zajic, Steven Amrein and Harald Dobnig

De Pascale and Quraishi raise a very important topic: how can we best assess an individual’s vitamin D status? The answer is that no one knows; not in healthy persons, and even less so in critical illness.

Certainly the intricate interplay of calcium and phosphate homeostasis, parathyroid hormone, fibroblast growth factor-23, and vitamin D metabolites is profoundly disturbed in critical illness, and particularly in sepsis. For a long time, total serum 25(OH)D was considered to best reflect an individual’s vitamin D status [6]. Recently, this choice of marker has been questioned because polymorphisms in the vitamin D binding protein gene seem to have important consequences on bioavailable 25(OH)D concentrations [7]. Moreover, vitamin D status at a tissue level is not measurable and the importance of circulating 1,25-dihydroxyvitamin D is unclear, although an association with mortality is known [8]. All of these considerations are true in critical illness, but there are even more potential confounders such as inflammation and acute fluid loading that may affect vitamin D concentrations.

In our retrospective cohort, we were partially able to adjust for these factors [1]. Unfortunately, vitamin D binding protein was not routinely measured, and thus we are unable to reanalyze our data to answer the very interesting question of whether an association of bioavailable 25(OH)D concentrations with clinically important outcomes is present. We agree that it may be important not only to correlate clinical outcomes with total 25(OH)D status, but also to consider respective changes in the bioavailable fraction. Besides prospective studies on how to assess vitamin D deficiency in this special setting, it will be crucial to perform large, well-planned multicenter randomized controlled trials to evaluate the effects of vitamin D on clinical outcomes, taking into account the important basic rules regarding the design for clinical studies on nutrient effects [9].

## Abbreviations

25(OH)D: 25-hydroxyvitamin D

## Competing interests

The authors declare that they have no competing interests.
